# Sex Differences in Exercise-Training-Related Functional and Morphological Adaptation of Rat Gracilis Muscle Arterioles

**DOI:** 10.3389/fphys.2021.685664

**Published:** 2021-07-12

**Authors:** Petra Merkely, Marcell Bakos, Bálint Bányai, Anna Monori-Kiss, Eszter M. Horváth, Judit Bognár, Rita Benkő, Attila Oláh, Tamás Radovits, Béla Merkely, Nándor Ács, György L. Nádasy, Marianna Török, Szabolcs Várbíró

**Affiliations:** ^1^Department of Obstetrics and Gynecology, Semmelweis University, Budapest, Hungary; ^2^Department of Physiology, Semmelweis University, Budapest, Hungary; ^3^Institute of Clinical Experimental Research, Semmelweis University, Budapest, Hungary; ^4^Heart and Vascular Center, Semmelweis University, Budapest, Hungary

**Keywords:** skeletal muscle arteriole, athlete’s arteriole, swim-training, sex difference, *in vitro* arteriography

## Abstract

**Background:**

The cardiovascular effects of training have been widely investigated; however, few studies have addressed sex differences in arteriolar adaptation. In the current study, we examined the adaptation of the gracilis arterioles of male and female rats in response to intensive training.

**Methods:**

Wistar rats were divided into four groups: male exercise (ME) and female exercise (FE) animals that underwent a 12-week intensive swim-training program (5 days/week, 200 min/day); and male control (MC) and female control (FC) animals that were placed in water for 5 min daily. Exercise-induced cardiac hypertrophy was confirmed by echocardiography. Following the training, the gracilis muscle arterioles were prepared, and their biomechanical properties and functional reactivity were tested, using pressure arteriography. Collagen and smooth muscle remodeling were observed in the histological sections.

**Results:**

Left ventricular mass was elevated in both sexes in response to chronic training. In the gracilis arterioles, the inner radius and wall tension increased in female animals, and the wall thickness and elastic modulus were reduced in males. Myogenic tone was reduced in the ME group, whereas norepinephrine-induced vasoconstriction was elevated in the FE group. More pronounced collagen staining was observed in the ME group than in the MC group. Relative hypertrophy and tangential stress of the gracilis arterioles were higher in females than in males. The direct vasoconstriction induced by testosterone was lower in females and was reduced as an effect of exercise in males.

**Conclusion:**

The gracilis muscle arteriole was remodeled as a result of swim training, and this adaptation was sex dependent.

## Introduction

The human body adapts to regular sports activities, such as adaptation processes, which appear in almost all parts of the body and at different levels of organization (Shephard et al., 2017; Fujita et al., 2019; Lacombe et al., 2019; Rosero et al., 2019). During sports activity, the workload of the cardiovascular system is markedly altered. The sports-related adaptation of the vascular system is not unified throughout the whole structure; instead, it is dependent on the type of training, location, and size of the vessels (Green et al., 2017).

Power training (e.g., bodybuilding) is characterized by muscle tone elevation, increased total peripheral vascular resistance, and slightly increased cardiac output. In contrast, in endurance training (e.g., runners and swimmers), cardiac output, heart rate, and blood flow velocity increase significantly, vessels in the skeletal muscles dilate, and total peripheral vascular resistance decreases (Hepple et al., 1999; Szauder et al., 2015).

Training affects vessels in different tissues in a different manner. In vessels of skeletal muscles (involved in training), flow is transiently increased; this is in contrast to visceral vessels, where flow decreases during exercise. During physical activity, the arterial pressure can be elevated, causing a cyclic increase in vessel wall tension, leading to morphological, biomechanical, and functional adaptation of the vessel. Elevation of endothelial nitrogen-oxide synthase (eNOS) is a characteristic feature in response to increased cyclic wall tension and repetitive shear stress, and results in an increase in NO-dependent vasodilation (Backshall et al., 2015; Green et al., 2017; Green and Smith, 2018).

In the case of peripheral musculocutaneous artery branches, the diameter of the vessel increases during a single exercise bout, and the vessel wall becomes thinner. However, the dilated vessel caliber is not always observable at rest (Green et al., 2012, 2017). There are conflicting results regarding the functional sport-related adaptation of peripheral vessels. In several studies, vascular reactivity increased, especially flow-mediated vasodilatation; this phenomenon has been described in both acute and chronic exercise settings (Atkinson et al., 2015; Ramos et al., 2015; Landers-Ramos et al., 2016). An earlier study that investigated short-term treadmill training adaptation of the male rat gracilis arteriole found a slight elevation in myogenic tone, no change in norepinephrine-induced tone, and a slight drop in adenosine-induced relaxation, while the acetylcholine and L-arginine (a precursor of NO) dilation degree substantially increased (Sun et al., 1994). Furthermore, short-term training increased the sensitivity of the endothelial cells to shear stress, resulting in an elevated dilation response (Koller et al., 1995). However, Green et al., reported the so-called “athlete paradox,” in which the initial improvement in the endothelial function caused by regular sport activities is later followed by a return to the original values due to further structural adaptation. In this scenario, there were no significant differences between the vascular functions of the trained and control vessels, following an extended exercise regime (Green et al., 2012).

The importance of sex differences in the study of sport-related adaptation of vessels is highlighted by the fact that there are significant sex-related differences in the incidence of cardiovascular diseases, which diminish only after menopause. Indeed, the protective effect of estrogen appears to be a decisive factor. In earlier studies, we investigated the morphological, biomechanical, and functional sex differences of coronary resistance vessels in a healthy state on the effects of hypertension and sports activities (Matrai et al., 2007; Mátrai et al., 2012; Török et al., 2020b). By studying coronary vessels, using an *in vitro* pressure myograph, we found that physical training in females led to an increase in contractility, while, in males, endothelium-related dilatation capacity was increased (Török et al., 2020b). This alteration observed in the male group, following long-term physical exercise, was not detected in postmenopausal women (Pierce et al., 2011). Based on these findings, we hypothesize that there are similar sex differences in sports-related adaptation of gracilis muscle arteries.

Blood flow in the arterioles of the gracilis muscle increases during both treadmill and swim training, although to a different extent. Extensor muscles are recruited more extensively and have a higher blood flow than flexors during treadmill exercise, while flexors are more strained during swimming (Laughlin et al., 1984).

In the current study, utilizing a relevant rodent model of exercise-induced myocardial hypertrophy, an extensive and lengthy swimming load was applied to rats, which resulted in substantial ventricular hypertrophy, as described before. Sex-dependent morphological, biomechanical, and functional remodeling of skeletal muscle arterioles was studied in the gracilis arterioles of the experimental animals.

## Materials and Methods

### Animals

Forty young adult male and female Wistar rats (12 weeks old) were housed at a constant temperature (22 ± 2°C) with a 12-h light-dark cycle. The rats were supplied with standard laboratory rat chow and tap water *ad libitum*. The experiments followed the regulations of the “Guide for the Care and Use of Laboratory Animals” by the National Institutes of Health (NIH Publication No. 86-23, revised 1996) and EU Directive 2010/63/EU. The program was approved by the Animal Care Committee of Semmelweis University and Hungarian authorities (permission number: PEI/001/2374-4/2015).

### Chemicals

Pentobarbital (Euthasol) from CEVA Santé Animale (Libourne, France) was used for anesthesia. The composition of the normal Krebs–Ringer solution (nKR) used for *in vitro* studies was as follows (in mmol/l): 119-mM NaCl, 4.7-mM KCl, 1.2-mM NaH_2_PO_4_, 1.17-MgSO_4_, 24-mM NaHCO_3_, 2.5-mM CaCl_2_, 5.5-mM glucose, and 0.0345 EDTA (ethylenediaminetetraacetic acid). The calcium-free Krebs–Ringer solution contained 92 NaCl, 4.7 KCl, 1.18 NaH_2_PO_4_, 20 MgCl_2_, 1.17 MgSO_4_, 24 NaHCO_3_, 5.5 glucose, 2 EGTA (ethylene glycol tetraacetic acid), and 0.025 EDTA. Salts were purchased from Reanal (Budapest, Hungary). Noradrenalin (NE) and testosterone (T) were obtained from Sigma-Aldrich (St. Louis, MO, United States). The frozen aliquots were diluted daily.

### Intensive Swim Training Protocol

Following 7 days of acclimatization, the animals were randomly divided into four experimental groups: male control (MC; *n* = 9), male exercise (ME; *n* = 10), female control (FC; *n* = 10), and female exercise (FE; *n* = 11).

Exercised animals (ME and FE) were subjected to a long-term (12-week-long) swim training program, which led to physiological left ventricular (LV) hypertrophy, as previously described (Radovits et al., 2013). The animals were placed in a water tank with plain walls, divided into six lines filled with tap water at 30–32°C at the same time of day in all training sessions. The training lasted 12 weeks, with 5 days of swimming and 2 days of rest each week. The initial duration of the training was 15 min on the first day, which was increased by 15 min every 2 days, until reaching a maximum of 200 min/day, which was maintained throughout the experiment; the 200-min swimming load was reached at the beginning of the 6th week, and this load was maintained until the end of the 12th week. Control male and female animals were placed in the water tank for 5 min daily, 5 days/week, in parallel to the 12-week training program of the swim-trained rats. The general shape and body weight of the animals were monitored regularly.

### Echocardiography

Echocardiographic assessments were performed as described previously (Kovacs et al., 2015) to confirm exercise-induced hypertrophy. Briefly, the animals were anesthetized with isoflurane (1–2% isoflurane in 100% oxygen). The body temperature of the animals was maintained at 37°C, using a heating bench. The chest was shaved, and transthoracic echocardiographic examination was performed with the rats in the supine position by an expert investigator who was blinded to the experimental groups. Standard two-dimensional short-axis records were acquired (at the mid-papillary level), using a 13-MHz linear transducer (GE Healthcare, Horten, Norway), and conducted on a Vivid i Echocardiac Image Analysis System (GE, Healthcare, United States). Images were analyzed, using image analysis software (EchoPac v113, GE Healthcare). M-mode images were used to measure left ventricular anterior wall thickness (LVAWT) and left ventricular posterior wall thickness (LVPWT) in diastole (index: d) and systole (index: s), and LV end-diastolic LLVEDD) and LV end-systolic (LVESD) diameters were measured. LV volumes were estimated according to the Teichholz method (Teichholz et al., 1976). The computed parameters were as follows:

• Stroke volume (SV) = LVEDV – LVESV
• SV index = SV/body weight

### *In vitro* Pressure Arteriography of Gracilis Arterioles

At the end of the swimming training program, the rats were anesthetized with pentobarbital (45 mg/kg body weight, intraperitoneal). The right common carotid artery was cannulated, blood pressure was measured, the chest was opened, and a slit was cut on the right atrium. Then, the whole body of the animal was perfused with 150-ml nKR solution. The hearts were removed, and their weights were measured. Among the adductor muscles, the gracilis muscle was identified, and its arterioles were carefully prepared under a preparation microscope (Wild M3Z) and removed. We started the preparation of the arteriole by separating the vascular bed of its from the accompanying vein and connective tissues. The proximal part of the vessel was used for the *in vitro* vascular measurements, while the distal part of the same vessel was fixed and used for histology. The distal part of the isolated gracilis arteriole can be a bit narrower than the proximal, as it may give side branches (if present, side branches were cut off). There can be minor variations in the orientation of arteriole segments in the paraffin blocks due to their very small size and possible side branches. The excised arteriole was immersed in warm (37°C) oxygenized nKR solution in a glass-bottomed tissue bath (Experimetria, Hungary) and was cannulated at both ends with plastic microcannulas (approx. 100-μm diameter). For gas bubbling, a gas mixture of 20% O_2_, 5% CO_2_, and 75% N_2_ was used, maintained at a pH of 7.4. The volume of the bath was 12 ml, and the solution was changed at 2.8 ml/min, using a roller pump. Drugs were added to the bath to yield the required final concentration (superfusion was suspended for this period). The microcannulas were also filled with warmed, oxygenated nKR, and were connected to servo-controlled pumps (Living Systems, Burlington, VT, United States) to set the intraluminal pressure. The gracilis arterioles were extended to their normal *in situ* length and pressurized under no-flow conditions. The organ chamber was positioned on the stage of an inverted microscope (Leica), with a light path to enable visualization of the changes in the outer and inner diameters of the arteries. Magnified pictures of the pressurized segments were acquired, using a DCM 130 E camera. Analysis of the arterioles was performed offline, using a specific image-analysis software [ImageJ; National Institutes of Health (NIH), Bethesda, MA, United States]. Length calibration was performed, using a micrometer etalon (Wild, Heerbrugg, Switzerland).

The following protocol was used to study the biomechanical and contractile parameters of the arterioles. Arterioles were taken from rats and incubated in oxygenated Krebs–Ringer solution at 50 mmHg of intraluminal pressure for 30 min. Arterioles develop spontaneous contractions under such circumstances (Nyborg et al., 1987). Following equilibration, the steady-state diameter was measured. Then, pressure-diameter curves were obtained after two conditioning pressure cycles (0–100–0–100–0 mmHg). The pressure was elevated in increments of 10 mmHg from 0 mmHg to 100 mmHg, and inner and outer diameters were measured at each level. The segment underwent 10 min of incubation at 50 mmHg; then noradrenalin (NE) was added at cumulative concentrations (10^–8^, 10^–7^, and 10^–6^ M; each concentration lasted 10 min), and the diameters were measured. Thereafter, without washing out the NE, the pressure diameter curves were recorded repeatedly. The drug was washed out, and the diameter of the segments was measured again after 10 min of incubation at 50 mmHg. The effects of testosterone were tested at concentrations of 10^–8^ and 10^–6^ mol/L (5–5 min for equilibration). Finally, the solution was changed to a calcium-free KR solution, and, after 30 min of incubation at 50-mmHg pressure, diameter plots in the passive state of the smooth muscle were recorded from 0 mmHg in 10 mmHg steps up to 100 mmHg.

The biomechanical parameters were calculated as follows:

• Wall thickness, h = r_*o*_ – r_*i*_;
• Wall/lumen ratio, Q = h/d_*i*_;
• Wall stress, σ = P^∗^r_*i*_/h, according to the Laplace–Frank equation;
• An incremental tangential elastic modulus of the cylindrical segments;
• E_*inc*_ = [2r_*o*_r_*i*_^2*^ΔP)/((r_*o*_^2^ – r_*i*_^2^)^∗^Δr_*o*_];
• Incremental distensibility was D_*inc*_ = ΔV/VΔP.

where h is the wall thickness in μm, r_*o*_ and r_*i*_ are the actual values of the outer and inner radii in μm, d_*i*_ is the inner diameter in μm, P is the transmural (intraluminal) pressure, Δr_*o*_ is the alteration of the outer radius during a pressure rise of ΔP according to Cox (1974), and ΔV is the change in vessel lumen volume relative to the initial volume of V in response to a pressure change of ΔP. Lumen volumes were computed from the inner radii, assuming cylindrical symmetry.

The following parameters were calculated from the pressure-diameter data:

• Myogenic tone (%) = (r_*iCa*_^2+^_*free*_ – r_*inKR*_)/r_*iCa*_^2+^_*free*_^∗^100
• Constrictions to NE (%) = (r_*iCa*_^2+^_*free*_ – r_*iNE*_)/r_*iCa*_^2+^_*free*_^∗^100
• Testosterone contraction (%) = (r_*inKR*_ – r_*iT*_)/r_*inKR*_^∗^100

where r_*iCa*_^2+^_*free*_ and r_*inKR*_ are the inner radii measured in a calcium-free solution and in a normal Krebs–Ringer solution at the same pressure, and r_*iNE*_ and r_*iT*_ are the inner radii measured after noradrenaline and testosterone at the same pressure, respectively.

### Histology

Gracilis arteriole segments used for biomechanical measurements were placed in 4% neutral buffered formalin. The dehydrated and paraffin-embedded tissues were cut into 5-μm sections. The tissues were stained, using MOVAT pentachrome stain (Russell modification), resulting in black (nuclei and elastic fibers), yellow (collagen and reticular fibers), red (muscle and fibrin), and blue (ground substance and mucin) areas. Sections were photographed with a Nikon Eclipse Ni-U microscope with a DS-Ri2 camera (Nikon Minato, Tokyo, Japan) at 10× magnification. To evaluate the results, pentachrome staining was separated into individual color channels on the images, using ImageJ software. After converting the separated images to black and white, the degree of staining was determined, using the non-calibrated optical density (OD).

### Statistical Evaluation

GraphPad Prism 5 software (GraphPad Software, La Jolla, CA, United States) was used for statistical analysis. Values are expressed as means, with the standard error of the mean included. Normal distribution was tested, using the Shapiro–Wilk method. In the case of normal distribution, two-way analysis of variance (ANOVA) with Tukey’s *post hoc* test was performed. In the case of non-normal distribution, the Kruskal–Wallis test, with Dunn’s *post hoc* test, was performed. *P*-values < 0.05 were considered to indicate statistically significant differences.

## Results

### Physiological Alterations

#### Effect of Exercise on the Body Weight and the Heart Weight/Body Weight Ratio of Rats

Body mass significantly increased during the 12-week observation period, but body mass elevation was significantly lower in the trained male group than in the sedentary male group. No difference was detected regarding body mass of the FC and FE groups (Table 1). The heart weight/body weight ratio measured postmortem was significantly increased in the exercised groups of both sexes compared with the control groups.

**TABLE 1:** The echocardiographic data of the experimental groups.

Variable	Male control	Male exercised	Female control	Female exercised
**Basic characteristics**				
Body weight^1^ (g)	309 ± 8	298 ± 5	210 ± 3^§^	214 ± 4^#^
Body weight^2^ (g)	496 ± 12	431 ± 9*	290 ± 4^§^	289 ± 4^#^
Heart weight/body weight (g/kg)	3.36 ± 0.05	3.95 ± 0.11*	3.79 ± 0.05^§^	4.49 ± 0.13^$#^
**Echocardiographic data**				
SV (μl)	245 ± 9	271 ± 6*	185 ± 6^§^	213 ± 9^$#^
SV index (μl/g)	0.49 ± 0.02	0.62 ± 0.02*	0.64 ± 0.01^§^	0.74 ± 0.03^$#^
LVAWTd (mm)	2.00 ± 0.01	2.17 ± 0.08*	1.93 ± 0.03	2.13 ± 0.04^$^
LVAWTs (mm)	3.25 ± 0.06	3.77 ± 0.10*	3.17 ± 0.07	3.45 ± 0.10^$#^
LVPWTd (mm)	1.87 ± 0.05	2.03 ± 0.04*	1.86 ± 0.04	1.92 ± 0.03^#^
LVPWTs (mm)	3.10 ± 0.06	3.30 ± 0.10*	2.80 ± 0.06^§^	3.01 ± 0.06^$#^

*BW^1^, body weight at the start of the training program; BW^2^, body weight at week 12; SV, stroke volume; LVAWT, left ventricular anterior wall thickness; LVPWT, left ventricular posterior wall thickness; d, diastole; s, systole. ^∗^*P* < 0.05 male control vs. male exercised. ^[*dollar*]^*P* < 0.05 female control vs. female exercised. ^§^*P* < 0.05 male control vs. female control. ^#^*P* < 0.05 male exercised vs. female exercised.*

#### Sex Differences in the Body Weight and the Heart Weight/Body Weight Ratio of Rats

Males of similar ages had higher body weights than females in both the sedentary and trained groups. Some differences in the advantage of males remained in the trained group despite less extensive weight gain during exercise in these animals. The heart weight/body weight ratio measured postmortem was significantly higher in female animals (FC and FE) than in males (MC and ME) (Table 1).

### Echocardiography

#### Effect of Exercise on Heart Morphology and Function

The echocardiographic data are shown in Table 1. Stroke volume values in the ME and FE groups were significantly higher than those in the corresponding sedentary animals. Furthermore, the SV index was significantly increased in both male- and female-exercised rats. At the end of the exercise training protocol, the left ventricular wall thickness values were significantly higher in the exercised rats. These data clearly indicate significant left ventricular hypertrophy both in male- and female-exercised animals.

#### Sex Differences in Heart Morphology and Function

No significant difference was found in LV wall thickness between the control male and female sedentary groups, with the exception of LVPWTs, which were lower in females. The stroke volume was lower in female rats than in male rats in both the control and exercised groups. In contrast to SV, the SV index was significantly higher in females in both the control and exercise groups. The LVAWTs, LVPWTd, and LVPWTs were significantly smaller in female-exercised rats than in exercised-male rats.

### Morphological and Biomechanical Parameters of Gracilis Arterioles

#### Effect of Exercise on Morphological and Biomechanical Parameters of Gracilis Arterioles

**FIGURE 1:**
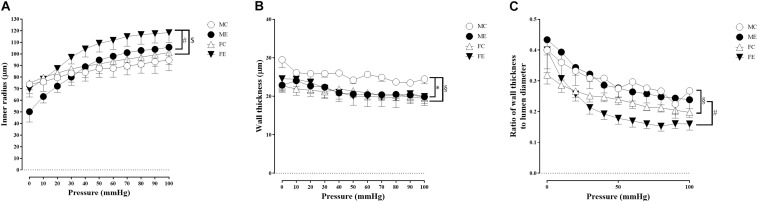
Morphological parameters of arteries from the gracilis muscle. **(A)** Inner radius of the gracilis muscle arterioles from the MC, ME, FC, and FE animals. The values of the inner radius as a function of intraluminal pressure measured under passive conditions (in calcium-free Krebs solution). The inner radius was increased in female exercised rats compared to FC and ME animals. Data are expressed as the mean (SEM) values. The significance levels of two-way ANOVA and Tukey’s *post hoc* tests between the four groups are shown. ^[*dollar*]^*P* < 0.05 FC vs. FE; ^#^*P* < 0.05 ME vs. FE. **(B)** Wall thickness of the gracilis muscle arterioles from MC, ME, FC, and FE animals. The values of the wall thickness as a function of intraluminal pressure measured under passive conditions (in calcium-free Krebs solution). The wall thickness was decreased in the ME group compared to the MC group, and this value was higher in MC rats than in FC animals. Data are expressed as the mean (SEM) values. The significance levels of two-way ANOVA and Tukey’s *post hoc* tests between the four groups are shown. ^∗^*P* < 0.05 MC vs. ME; ^§^*P* < 0.05 MC vs. FC. **(C)** Wall thickness to lumen diameter ratio of the gracilis muscle arterioles from MC, ME, FC, and FE animals. The wall thickness to lumen ratio was significantly smaller in FC and FE rats than MC and ME rats. Data are expressed as the mean (SEM) values. The significance levels of two-way ANOVA and Tukey’s *post hoc* tests between the four groups are shown. ^§^*P* < 0.05 MC vs. FC; ^#^*P* < 0.05 ME vs. FE.

**FIGURE 2:**
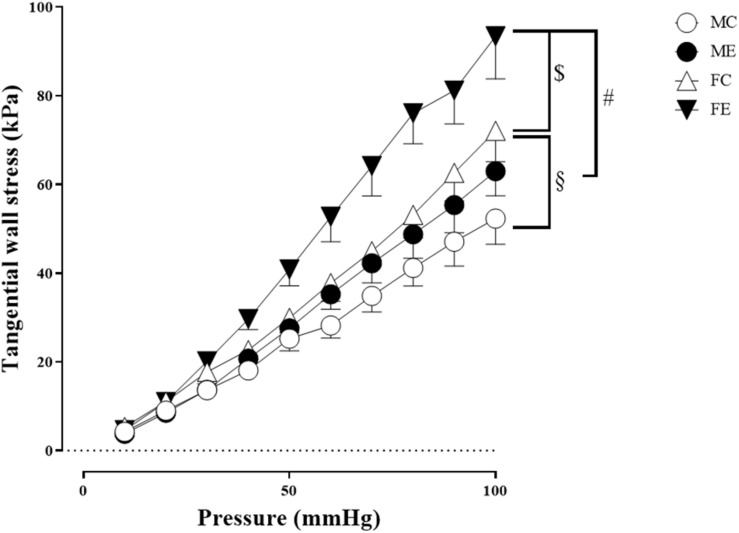
Tangential wall stress as a function of intraluminal pressure measured in passive conditions of the gracilis muscle arterioles from the MC, ME, FC, and FE animals. The tangential wall stress was significantly increased in the FE rats compared with the controls. In addition, this value was significantly higher in the FC and FE rats than in the MC and ME rats. The data are expressed as the mean (SEM) values. The significance levels of two-way ANOVA and Tukey’s *post hoc* tests between the four groups are shown. ^[*dollar*]^*P* < 0.05 FC vs. FE; ^§^*P* < 0.05 MC vs. FC and ^#^*P* < 0.05 ME vs. FE.

**FIGURE 3:**
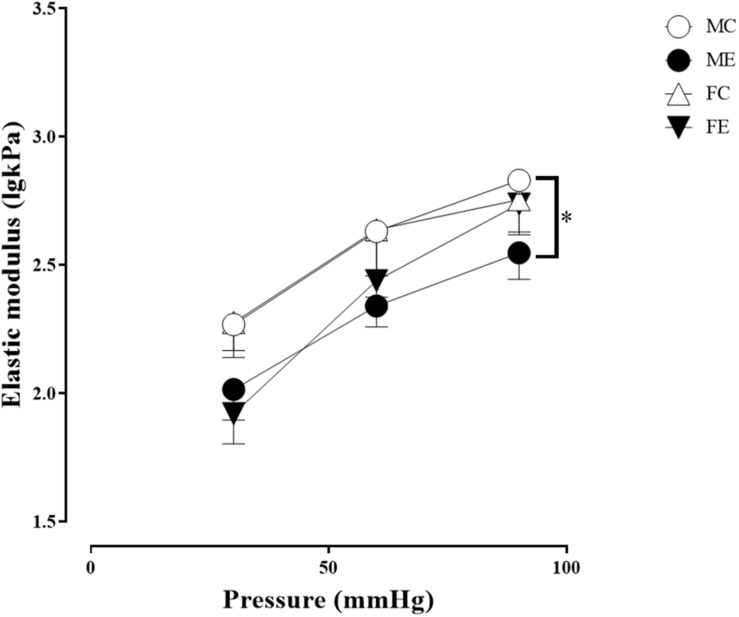
Tangential elastic modulus as a function of intraluminal pressure measured in passive conditions of the gracilis muscle arterioles from the MC, ME, FC, and FE animals. As a result of the exercise, the elastic modulus was significantly smaller in the ME rats than in the MC rats. The data are expressed as the mean (SEM) values. The significance levels of the two-way ANOVA and Tukey’s *post hoc* tests between the four groups are shown. ^∗^*P* < 0.05 MC vs. ME.

**FIGURE 4:**
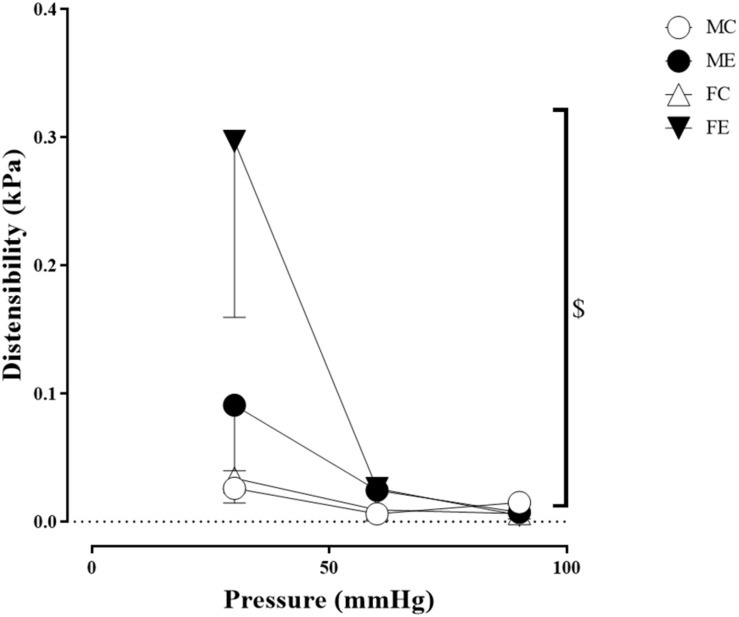
Distensibility as a function of intraluminal pressure measured under passive conditions of the gracilis muscle arterioles from the MC, ME, FC, and FE animals. Distensibility increased in the FE rats compared with the FC rats. The data are expressed as the mean (SEM) values. The significance levels of the two-way ANOVA and Tukey’s *post hoc* tests between the four groups are shown. ^$^*P* < 0.05 FC vs. FE.

Despite the fact that all harvested arteriolar segments were anatomically and morphologically identical at preparation, there was a significant difference in the inner radius of the vessels between the groups in the relaxed state. The inner radius was significantly higher in the female-exercised animals than in the control females (Figure 1A). The wall thickness was significantly decreased in the male-exercised animals compared with that in the male control rats (Figure 1B). Moreover, as an effect of exercise, there was no significant difference in the wall thickness to the lumen diameter ratio between the control and exercised rats in both the male and female groups (Figure 1C). Tangential wall stress was significantly higher in the trained females than in their sedentary counterparts (Figure 2). The elastic modulus was significantly reduced in the male-exercised rats compared with that in the male control animals (Figure 3). Following the exercise regime, the females showed significantly increased distensibility (Figure 4).

#### Sex Differences in Morphological and Biomechanical Parameters of Gracilis Arterioles

Importantly, the inner radius elevation could only be observed in the exercised females. While there was no significant difference in the inner radius between the sedentary males and females, the exercised females had wider gracilis arterioles than the males (Figure 1A). Moreover, the wall thickness was significantly lower in the female control group compared with the male control group, and this difference was diminished in the trained animals (Figure 1B). In the female rats (the FC and FE groups), we observed a significantly smaller wall thickness-to-lumen diameter ratio than those observed in the male rats (Figure 1C). Tangential stress increased in the female animals as an effect of training, but no such change was observed in the male specimens, and the tangential stress was significantly higher in the female animals (FC and FE) than in the male rats (MC and ME) (Figure 2). Moreover, training-induced reduction of the elastic modulus occurred only in the males (Figure 3), while another elastic parameter, incremental distensibility, increased only in the females (Figure 4). No further difference between the males and the females, either in the sedentary or trained animals, was identified in any of the elastic parameters (Figures 3, 4).

### Contractility Parameters for Gracilis Arterioles

#### Effect of Exercise on Contractility Parameters for Gracilis Arterioles

**FIGURE 5:**
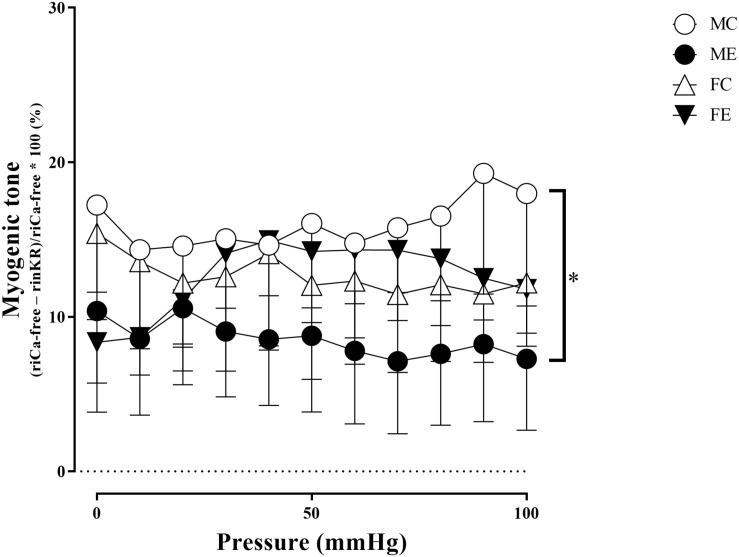
Myogenic tone as a function of intraluminal pressure measured under passive conditions of the gracilis muscle arterioles from the MC, ME, FC, and FE animals. As a result of the exercise, the myogenic tone was significantly smaller in the ME rats than in the MC rats. The data are expressed as the mean (SEM) values. The significance levels of the two-way ANOVA and Tukey’s *post hoc* tests between the four groups are shown. ^∗^*P* < 0.05 MC vs. ME.

**FIGURE 6:**
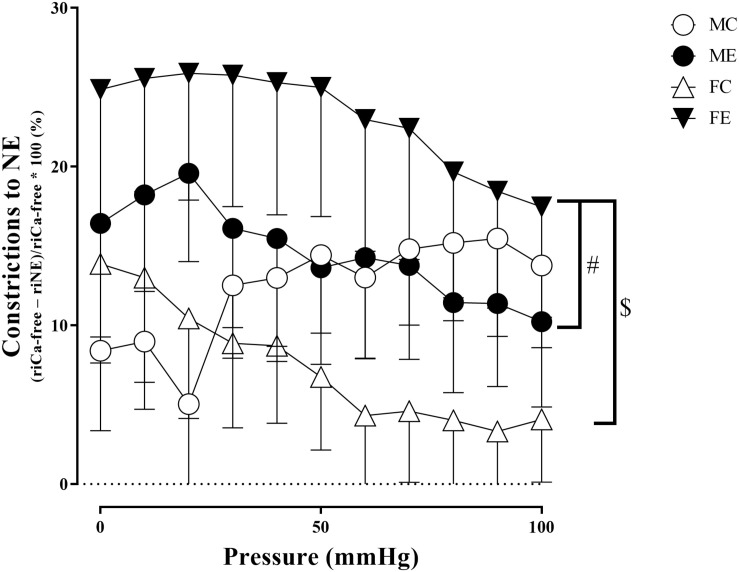
Constrictions to NE (relative difference of the maximally relaxed and maximally contracted radius) as a function of intraluminal pressure measured in passive conditions of the gracilis muscle arterioles from the MC, ME, FC, and FE animals. As a result of norepinephrine, the constriction was significantly higher in the FE rats compared with the FC and ME rats. The data are expressed as the mean (SEM) values. The significance levels of the two-way ANOVA and Tukey’s *post hoc* tests between the four groups are shown. ^$^*P* < 0.05 FC vs. FE and ^#^*P* < 0.05 ME vs. FE.

**FIGURE 7:**
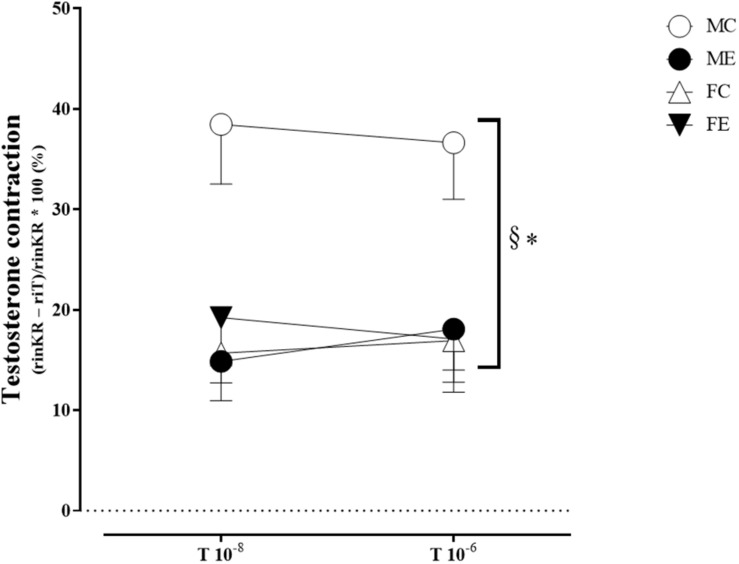
Testosterone contraction of the segments in 10^–8^ and 10^–6^ M at 50 mmHg from the MC, ME, FC, and FE animals. The testosterone contraction was significantly lower in the females than in the males in the control groups. As a result of the exercise, the testosterone contraction decreased in the male groups. The data are expressed as the mean (SEM) values. The significance levels of the two-way ANOVA and Tukey’s *post hoc* tests between the four groups are shown. ^∗^*P* < 0.05 MC vs. ME and ^§^*P* < 0.05 MC vs. FC.

The myogenic tone of the gracilis muscle arterioles in the male exercise group was significantly lower than that in the vessels harvested from MC rats (Figure 5). In terms of changes in contractility (NE constriction), the extent of contraction of the segments (relative difference of maximally relaxed and maximally contracted radius) was increased in the females, following the swimming training (Figure 6). The level of testosterone contraction was significantly decreased in the ME group, approaching that observed in the female animals (Figure 7).

#### Sex Differences in Contractility Parameters for Gracilis Arterioles

A training-induced reduction in myogenic tone was only observed in the male animals. Sex differences in myogenic tone did not reach statistical significance in either the control or trained groups (Figure 5). Training elevated norepinephrine vasoconstriction in the females, but not in the males; as a result, the trained females exerted more effective vasoconstriction than the trained males (Figure 6). Contraction to testosterone was significantly lower in the FC rats than in the MC rats. This difference diminished after the training (Figure 7).

### Histology Changes

#### Effect of Exercise on Histological Sections

**FIGURE 8:**
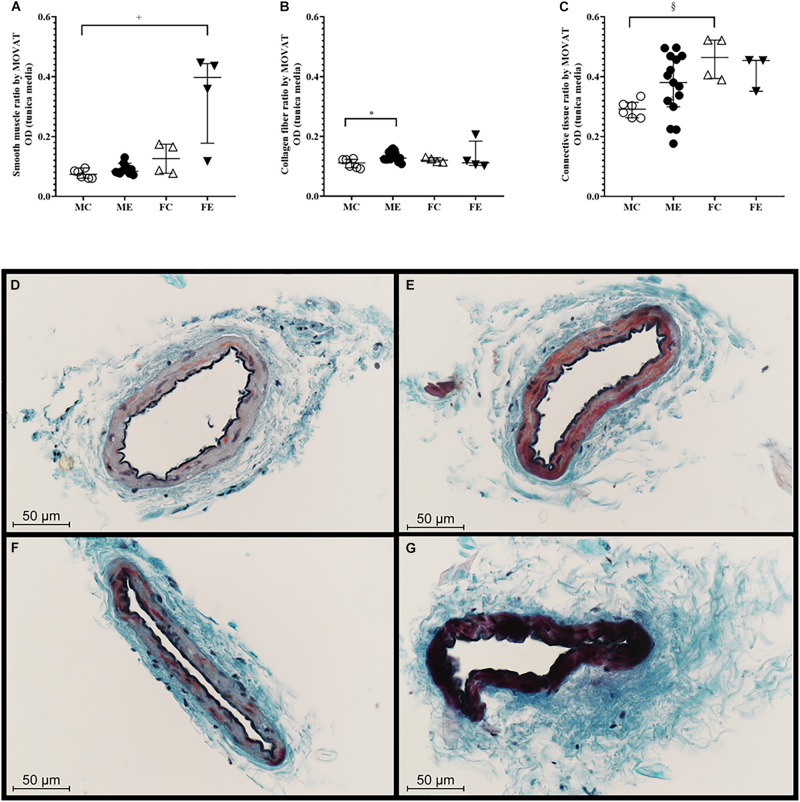
MOVAT staining. **(A)** Smooth muscle staining intensity of segments from the MC, ME, FC, and FE animals. Smooth muscle density was significantly higher in the FE rats than in the MC rats. **(B)** Collagen staining intensity of the segments from the MC, ME, FC, and FE animals. The density of the collagen was significantly higher in the male exercised group compared with the male control rats. **(C)** Connective tissue staining of the segments from the MC, ME, FC, and FE animals. **(D)** Representative staining in the MC rats. **(E)** Representative staining in the ME rats. **(F)** Representative staining in the FC rats. **(G)** Representative staining in the FE rats. The density of connective tissue was significantly higher in the female control group than in the male control group. The data are expressed as the median (interquartile ranges) values. The significance levels of Kruskal–Wallis test and Dunn’s *post hoc* tests between the four groups are shown. ^+^*P* < 0.05 MC vs. FE; *P < 0.05 MC vs. ME and ^§^*P* < 0.05 MC vs. FC.

The medial smooth muscle content was not significantly altered by the training (Figure 8A). The density of collagen was higher in the ME group than in the MC group (Figure 8B). No training-induced alteration in the overall medial connective tissue content was observed (Figure 8C). Figures 8D–G show representative MOVAT-stained sections of the gracilis arterioles prepared from the four groups.

#### Sex Differences in Histological Sections

There was a significant difference between the male-control and female-exercised animals; the FE rats demonstrated more intense muscle staining in their tunica media than the MC rats (Figure 8A). Moreover, collagen staining increased in the males in response to training, and connective tissue staining was significantly higher in the female control group than in the male control group (Figure 8C).

## Discussion

In the current study, we examined the effects of exercise training on gracilis muscle arterioles in the male and female rats. To the best of our knowledge, this is the first study to investigate sex differences in a swim-training model of skeletal muscle arteriole biomechanics and pharmacology. The major findings of our investigation can be summarized as follows: (1) The massive exercise load of our protocol is demonstrated by the development of an “athlete-type” heart in our animals. (2) Vascular adaptation in the trained muscle showed sex differences in that the females showed an increase in the supplying arteriolar diameter (dilatation), while the males demonstrated thinning of the arterial wall (morphological remodeling of the wall). (3) Wall tension increased in the exercised females as a result of vascular dilatation, while, in males, the elasticity of the vessels was increased. (4) As a result of swimming, the adaptation range (the range between maximum contraction and maximum relaxation—it determines the ability for vasomotion) of the gracilis arteriole widened during both contraction and dilatation, but this proved to be dependent on sex; myogenic tone decreased in the males, while the maximum contraction capacity increased in the females. (5) Our histological findings also showed sex differences in response to physical exercise. In the females, the smooth muscle content of arterioles increased, while in the males, there was more collagen in the media. These results confirm our theory that sex plays an essential role in the sport adaptation of skeletal muscle arterioles. Further studies may provide a basis for the implementation of individual/sex-optimized training programs.

### Effect of Strenuous Training on the Heart, and Morphology and Biomechanics of Gracilis Arterioles

Echocardiographic examinations were performed to test the training status of the animals. In accordance with our previous results and published data in the literature, 12 weeks of the swim training leads to substantial myocardial hypertrophy in both males and females (Olah et al., 2019; Török et al., 2020b).

Although training adaptation of peripheral vessels has been examined in several earlier studies, part of these observations is focused on larger (conduit) arteries (Andaku et al., 2020). Alterations of small arteries in response to training have been studied earlier in some publications (Jasperse and Laughlin, 1999; Laughlin et al., 2004; Spier et al., 2004).

It is known that the walls of the peripheral arteries thicken and their diameter increases as a result of physical exercise (Green et al., 2012, 2017). Elevation of maximal diameter is not always present. While the diameter of arterioles of gastrocnemius muscle significantly increased as an effect of training both in young and elderly rats, no similar alterations in the soleus muscle arterioles could be observed (Spier et al., 2004). In a study of elite squash players, it was found that, in the non-dominant arm, the wall of the brachial artery became thinner, but its diameter remained unchanged, while, in the dominant arm, arterial wall thinning was accompanied by vascular dilatation (Thijssen et al., 2012). This phenomenon may be explained by the fact that the arteriole of the dominant arm receives higher shear stress for a longer period than the arteries in the non-dominant arm (Thijssen et al., 2012). Another explanation is that the vascular tone decreases due to training, leading to thinning of the wall (Amaral et al., 2000; Thijssen et al., 2012). In our recent research, we examined the morphological and biomechanical parameters of gracilis arterioles. In the exercised male animals, the inner vascular diameter remained unchanged, while the wall thickness decreased to the values observed in the females. However, the wall thickness-to-lumen ratio did not change significantly in either sex.

The effect of training the wall stress increased in the females, while no change was observed in the males; this may be caused by the enhanced vascular diameter, and the fact that the wall tension is directly proportional to the lumen radius of the vessel and inversely proportional to wall thickness. In the literature, both increases and decreases in vascular wall tension have been reported as effects of training in different vessels (Nualnim et al., 2011, 2012; Yuan et al., 2016). These contradictory results can be explained by the use of different study subjects, types of arteries, training protocols, and study methods. In this study, we used an *in vitro* pressure myograph to track the morphological parameters of vessels in their passive state, from which the biomechanical parameters were derived. Elastic parameters are connected to geometry and will be discussed in the later subchapter.

### Effect of Strenuous Training on the Gracilis Arterioles

The functional adaptation caused by training was not uniform along the vascular system. The vessel segments have different functions, depending on their size, location in networks, and lying in different vascular beds. Small vessels and arterioles contract spontaneously if common physiological conditions are present; a spontaneous or myogenic tone can be observed. There are contradictory findings in the literature concerning the effect of chronic exercise on myogenic tone, with studies showing increased, decreased, and unchanged basal arterial tone (Meredith et al., 1990; Amaral et al., 2000; Sun et al., 2002; Korzick et al., 2004; Green et al., 2012). In older rats (28–30 weeks old), there were no significant differences in the myogenic tone of the gracilis arterioles in either control or trained male animals (Sun et al., 2002). In contrast, after a month of training, the total peripheral resistance index decreased in the healthy young men (Meredith et al., 1990). Sun et al., investigated the effects of a moderate training program in male rats, using a pressure arteriography on gracilis arterioles. They found a slight elevation in myogenic tone, and no significant difference in norepinephrine tone (Sun et al., 1994). It is interesting that the extent of myogenic tone can depend upon the type of the vessel studied. In interval sprint-trained rats, arterioles prepared from the gastrocnemius muscle had higher myogenic tone than feed arteries of the same muscle (Laughlin et al., 2004). Interval sprint training elevates the spontaneous tone of arterioles, but their myogenic reactivity is the same as that of control arterioles, keeping diameter almost constant despite alterations in intraluminal pressure (Laughlin et al., 2004). These observations are in contrast to those of our study, in which, following a 12-week strenuous training program, the myogenic vessel tone decreased in the young adult male rats and remained unchanged in the female rats. Moreover, in the females, the norepinephrine tone was elevated in the present study. Another interesting observation is that, while in skeletal muscle arterioles of the rat, there is an elevation of alpha-adrenergic and endothelin-1 mediated contractions with age (Donato et al., 2005, 2007); in elderly rats, training can reduce alpha-adrenergic vasocontraction (Donato et al., 2007), not affecting at the same time endothelin-1 vasoconstriction in soleus muscle and gastrocnemius arterioles (Donato et al., 2005).

In our studies, the vasoconstriction-lowering effect of training could be observed in young male rats. There is an observation according to vascular adaptation in trained elderly animals can be different in metabolically different types of muscle. High oxidative portions of gastrocnemius (red gastrocnemius) are low perfused, while low-oxidative portions of gastrocnemius (white gastrocnemius) are relatively highly perfused when compared with young animals (Behnke et al., 2012). Endurance training in elderly animals improves the matching of oxygen delivery to an oxidative capacity of the muscle; it increases vascular conductance and blood flow in red gastrocnemius, while moderating these parameters in the white gastrocnemius (Behnke et al., 2012). A further important observation was that, in the young animals, training elevated the total number of arteries perforating the gastrocnemius muscle but left the cross-sectional area of the feed artery unaltered, while, in the elderly animals, in an opposite manner, the number of perforating arteries was unaltered while the diameter of the feed artery increased (Behnke et al., 2012). Potential age-induced differences in arteriolar adaptation are outside the scope of the present study, forming one of its limitations.

The length and extent of training can explain the difference between these observations. We found that 12 weeks of strenuous work is sufficient for geometrical and histological wall remodeling. Moreover, even the sympathetic innervation of these vessels could have changed when sufficient time was provided.

Testosterone has an acute vasoactive effect; the strength of which varies according to species and vessel types (Perusquía et al., 2012; Pál et al., 2019). In our recent experiment, testosterone was added to the organ bath-induced contraction of gracilis vessels. The training reduced testosterone contraction in the males, but no significant alteration in the females was observed (see the next subsection).

### Sex Differences in Heart Morphology and Function

The absolute cardiac parameters measured by echocardiography were significantly lower in the females but were higher when adjusted to the lower weight of the female animals. Based on the literature, the Akt-protein may play a role in the enhanced relative cardiac hypertrophy of females. In our previous study, phosphorylation of Akt increased in both sexes as a result of exercise but was more pronounced in the females (Olah et al., 2019). In an earlier study from our group, we found sex differences in the activation of extracellular signal-regulated kinase 1/2 (ERK1/2), mammalian target of rapamycin (mTOR), and S6 (ribosomal protein), and in the ratio of α/β-MHC (myosin heavy chain) (Olah et al., 2019). Similar results were reported not only in animal models but also in male and female athletes. Similar to our animal observations, women have smaller absolute cardiac sizes, although the values indexed to the body surface area are greater in sportswomen than in men (D’Ascenzi et al., 2020).

### Sex Differences in the Morphology and Biomechanics of Gracilis Arterioles

Significant sex differences were observed in the control group with respect to the wall thickness. In exercised female animals, we found an increase in the inner vascular diameter, while the wall thickness remained unchanged. This increase was more pronounced than in the case of the exercised males. The male controls had thicker vascular walls, the trained males had narrower vascular diameters, and the wall thickness/lumen ratio was higher in the males than in the females in both the control and trained groups. The effect of sex differences on the vascular wall thickness/lumen ratio in gracilis and other arterioles was studied in previous studies on hypertension (Amaral et al., 2000; Amaral and Michelini, 2011). Similar to our observations, chronic exercise did not significantly affects the wall/lumen ratio in the male Wistar–Kyoto rats. However, chronic exercise restored the normal value in the spontaneous hypertensive (SHR) rats, but not in the female SHRs (Amaral et al., 2000; Amaral and Michelini, 2011). Green et al., found that there were no sex-related differences in the wall thickness of the brachial and popliteal arteries in younger and older sportsmen, while the vessel diameter was larger (and, therefore, the wall thickness/lumen ratio was lower) in men. Furthermore, as a result of a 12- and 24-week training programs, the wall/lumen ratio was shown to decrease in both sexes (Green et al., 2010). Unsurprisingly, the type of training has an effect on the wall thickness/lumen ratio; resistance training causes a decrease in the wall thickness/lumen ratio in brachial arteries, while, in aerobic training, it remains unchanged in patients with chronic heart failure (Maiorana et al., 2011).

The observed higher wall thickness/lumen ratio in our male animals resulted in lower vascular wall tension of the gracilis vessels in both the control and trained groups.

The distensibility and elastic modulus are parameters that describe the elasticity of the vessels. Higher distensibility and lower elastic modulus result in greater vascular elasticity. The elasticity of the gracilis vessels increased as a result of training, but it showed sex-related differences. In the males, the elastic modulus decreased, whereas, in the females, the distensibility increased at low pressures. Similar to our findings, a decrease in elastic modulus and an increase in distensibility of resistance coronary arteries were observed in the rats trained on a treadmill (Szekeres et al., 2018). The increase in the vascular parameters related to elasticity is advantageous in terms of hemodynamic adaptation. During exercise, flow in the moving skeletal muscles increases periodically; therefore, the gracilis vessels may dilate more effectively as a result of their adaptation, resulting in improved tissue perfusion.

### Sex Differences in Arteriolar Contractile Function

Similar results have been found in a previous study on coronary arterioles of swimming rats, in which the myogenic tone of trained male rats was significantly lower than that of females (Török et al.,2020a,b). In contrast, the maximum contraction caused by noradrenaline was significantly increased in our trained female animals. Some sex differences in the contraction of muscle arteries following training have already been examined, but the results in the literature are inconsistent (Just and DeLorey, 2017; Samora et al., 2019). Just and DeLorey (2017) found an increased vasoconstriction capacity in females in a steady state compared with males, but the difference disappeared during muscle contraction. However, Laughlin et al. (2001) found that, following chronic training, endothelin-1 contraction increased, while KCl and norepinephrine contractions remained unchanged in the gracilis arterioles of a male miniature swine. Soleus feed arteries of male rats responded with dose-dependent contractions to norepinephrine; no difference between trained and sedentary animals could be observed (Jasperse and Laughlin, 1999). Gastrocnemius feed arteries were more sensitive to phenylephrine in the control than in the trained animals (Laughlin et al., 2004).

In aortic rings of rats after 10 weeks of training, there was a reduced norepinephrine sensitivity. It could be observed only after 4–10 weeks of training. Removal of endothelium diminished that reduced sensitivity, proving its endothelial origin (Spier et al., 1999).

Based on this and on our observations, it may be considered that, as a result of training, the vascular reactivity increases in both sexes; in trained males, relaxation is more pronounced, while, in trained females, higher contraction is observed. The regulatory effects of estrogen and testosterone may play a role in these effects, and further investigations are needed to explain the observed sex differences.

Substantial sex differences could be demonstrated in the acute vasoactive effect of testosterone; the most intensive contraction was found in the male control animals, which decreased to the level of the females as a result of exercise. One explanation for this phenomenon is that exercise increases the metabolism of testosterone to estrogen, which compensates for the vasoconstrictor effect of testosterone (Gharahdaghi et al., 2020; Moreau et al., 2020). In addition, the dominance of testosterone-related vascular effects can move toward relaxation. Testosterone affects vascular tone through NO-dependent vasorelaxation, prostanoid-dependent vasoconstriction, and relaxation (Chinnathambi et al., 2013), and the relative proportion of these effects may vary in response to exercise.

### Histological Remodeling

Our histological observations also showed sex differences in the training-induced histological remodeling of skeletal muscle arterioles. While females improve their smooth muscle ratio, male rats enhance the amount of collagen in the tunica media. The differences in the histological composition may be related to the observed differences in wall elasticity and contractility.

### Study Limitations

One limitation of our experiments is that they have been performed on relatively young animals, which makes it difficult to adapt to mammals with a slower life cycle, or to humans, where circulatory problems arise mostly in advanced age. Further observations are needed to reveal the degree to which different sex hormones are responsible for the observed sex differences.

## Conclusion

As a result of a strenuous swimming exercise program, a peripherally located skeletal muscle arteriole, the gracilis arteriole adapts to physical activity; this adaptation shows sex differences: In the females, the wall thickness remained unchanged, and the diameter increased, while, in the males, the diameter remained the same, and thinning was observed in the vessel wall. In the males, the elastic modulus decreased due to exercise, while, in the females, increased distensibility was observed. Sport adaptation also appears in the altered contractility of the vessels, and we succeeded to identify substantial sex differences in the sport-induced alterations of basal and induced tones. In the males, the myogenic tone decreased, whereas the maximum contraction was enhanced in the females. The biomechanical and functional changes following chronic physical activity may be considered as physiological regulation. The observed processes ensure increased flow during physical activity, defense against increased wall stress during work dilation, and represent a convenient way to reduce blood flow during inactivity.

## Data Availability Statement

The original contributions presented in the study are included in the article/supplementary material, further inquiries can be directed to the corresponding author/s.

## Ethics Statement

The animal study was reviewed and approved by the Animal Care Committee of Semmelweis University and Hungarian authorities (Permission Number: PEI/001/2374-4/2015). Throughout the experiments, the regulations of the “Guide for the Care and Use of Laboratory Animals” by the National Institutes of Health (NIH Publication No. 86-23, revised 1996) and the EU Directive 2010/63/EU were followed.

## Author Contributions

PM: formal analysis, investigation, data curation, and writing—original draft, review, and editing. MB and AM-K: investigation, data curation, and writing—review and editing. BB, EH, RB, JB, and AO: investigation, formal analysis, data curation, and writing—review and editing. TR, BM, and SV: conceptualization, methodology, validation, writing—review and editing, and funding acquisition. NÁ: conceptualization, methodology, validation, and writing—review, and editing. GN: formal analysis, data curation, investigation, conceptualization, methodology, validation, writing—review and editing, and funding acquisition. MT: formal analysis, investigation, resources, data curation, writing—original draft, review, and editing, visualization, validation, and project administration. All authors contributed to the manuscript, and read and approved the final version of the manuscript.

## Conflict of Interest

The authors declare that the research was conducted in the absence of any commercial or financial relationships that could be construed as a potential conflict of interest.
